# Evolution of kinase polypharmacology across HSP90 drug discovery

**DOI:** 10.1016/j.chembiol.2021.05.004

**Published:** 2021-10-21

**Authors:** Albert A. Antolin, Paul A. Clarke, Ian Collins, Paul Workman, Bissan Al-Lazikani

**Affiliations:** 1 Department of Data Science, The Institute of Cancer Research, London SM2 5NG, UK; 2 Cancer Research UK Cancer Therapeutics Unit, The Institute of Cancer Research, London SM2 5NG, UK

**Keywords:** HSP90, drug discovery, polypharmacology, drug design, off-targets, computational, cancer, kinase, cross-pharmacology, pharmacology

## Abstract

Most small molecules interact with several target proteins but this polypharmacology is seldom comprehensively investigated or explicitly exploited during drug discovery. Here, we use computational and experimental methods to identify and systematically characterize the kinase cross-pharmacology of representative HSP90 inhibitors. We demonstrate that the resorcinol clinical candidates ganetespib and, to a lesser extent, luminespib, display unique off-target kinase pharmacology as compared with other HSP90 inhibitors. We also demonstrate that polypharmacology evolved during the optimization to discover luminespib and that the hit, leads, and clinical candidate all have different polypharmacological profiles. We therefore recommend the computational and experimental characterization of polypharmacology earlier in drug discovery projects to unlock new multi-target drug design opportunities.

## Highlights

• Characterization of kinase polypharmacology landscape for HSP90 inhibitors
• Ganetespib and luminespib display unique polypharmacology
• Kinase polypharmacology evolved during optimization to discover luminespib
• Earlier characterization of polypharmacology in drug discovery is recommended

## Graphical abstract

**Figure:**
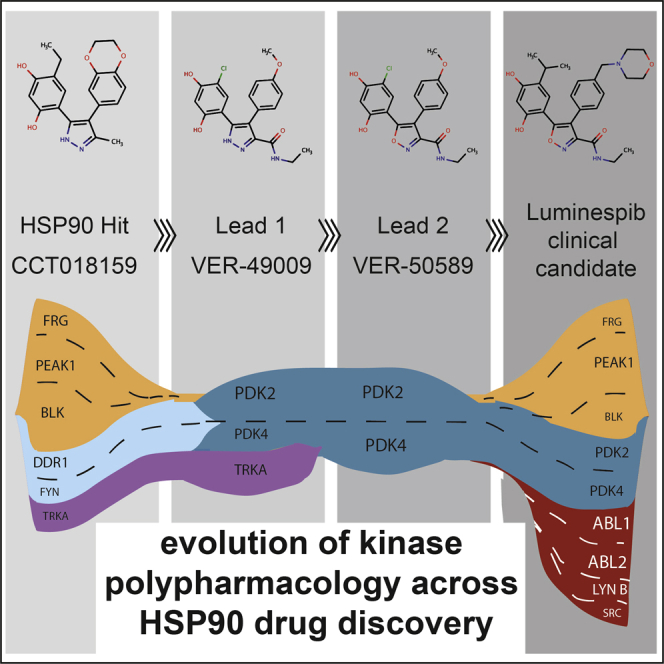

## Teaser

Polypharmacology is seldom comprehensively investigated during drug discovery. Antolin et al. describe the identification of polypharmacology for two HSP90 inhibitors and how it evolved during the discovery of luminespib, highlighting the importance of characterizing polypharmacology earlier in drug discovery.

## Introduction

It is widely accepted that most small molecules will interact with multiple molecular targets when exposed to complex biological systems and the term polypharmacology is commonly used to refer to this phenomenon (Blagg and Workman, 2017; Paolini et al., 2006). It is also widely acknowledged that off-targets can influence both drug efficacy and safety in the clinic (Antolin et al., 2016; Proschak et al., 2019). Moreover, polypharmacology of hit and lead compounds may inadvertently influence the direction and success of drug discovery projects. Despite this, polypharmacology is not being routinely explored as part of the drug discovery journey. Rather, to maximize cost-efficiency and maintain focus, potential off-targets are generally profiled only for very few compounds at late project stages.

Heat shock proteins (HSPs) are a group of molecular chaperones that are upregulated under stress to enable the correct folding of proteins (Butler et al., 2015; Chiosis et al., 2013; Schopf et al., 2017). The 90 kDa heat shock protein HSP90 is one of the most abundant HSPs and a key regulator of proteostasis in both physiological conditions and under stress (Butler et al., 2015; Schopf et al., 2017). Through the folding and stabilization of several hundred substrates, termed client proteins, HSP90 modulates many cellular processes beyond proteostasis, including signal transduction, DNA repair, and immune response, that are important in several diseases, such as cancer, neurodegenerative conditions, inflammation, and infection (Butler et al., 2015; Shrestha et al., 2016; Taipale et al., 2010; Workman, 2020).

**Figure 1:**
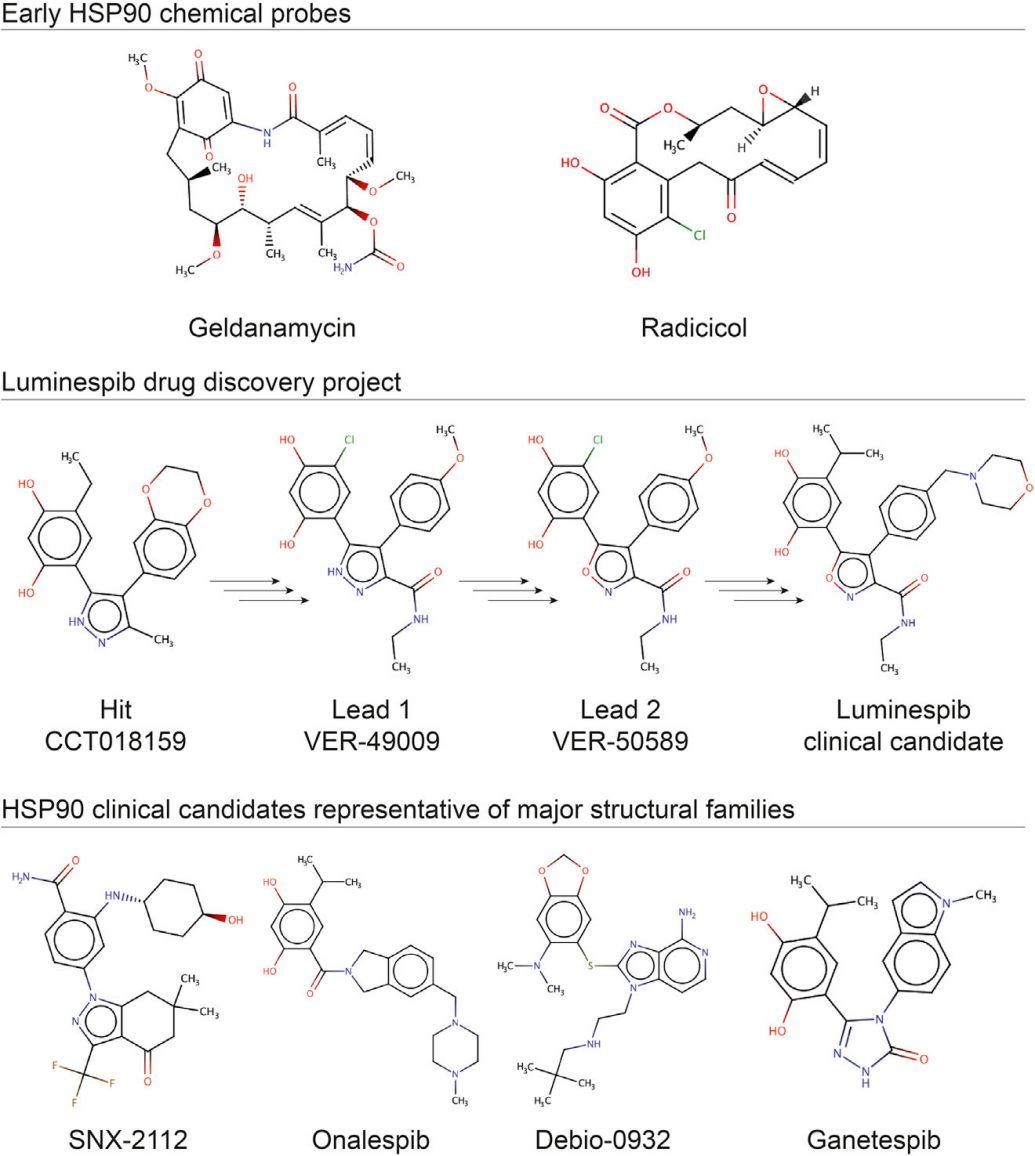
Chemical structures of selected HSP90 inhibitors

Thus, HSP90 became a widely pursued drug target (Schopf et al., 2017). HSP90 inhibitors in the clinic fall into two major lineages: (1) derivatives of the natural product geldanamycin and (2) non-natural product inhibitors. The first classes of synthetic small-molecule HSP90 inhibitors included purine (Shrestha et al., 2016) and resorcinol derivatives (the latter sharing this motif with the natural product radicicol; for chemical structures see Figure 1). Luminespib, ganetespib, and onalespib are the resorcinol derivatives that have advanced furthest in clinical trials (Butler et al., 2015; Koren and Blagg, 2020; Shrestha et al., 2016). Debio-0932, BIIB021, and PU-H71, are among the most clinically advanced purine derivatives (Workman, 2020). An additional class of HSP90 inhibitors harboring a benzamide moiety has been reported, SNX-2112 being one of the most clinically advanced compounds of this group (Biamonte et al., 2010).

The clinical development of HSP90 inhibitors to date has focused mainly on N-terminal ATP-site inhibitors for oncology indications. The first class of HSP90 inhibitors to be pursued clinically were geldanamycin derivatives (benzoquinone ansamycins). These provided the initial clinical proof-of-concept and validation of HSP90 as a cancer target but generally showed modest efficacy and were limited by unfavorable properties, particularly liver toxicity, most likely due to the quinone moiety (Banerji, 2009; Garcia-Carbonero et al., 2013). Second-generation synthetic HSP90 inhibitors solved some of the limitations of the first generation and again showed clinical activity (Pillai and Ramalingam, 2018). The most promising responses were seen in HER2-positive breast cancer and in non-small cell lung cancer (NSCLC) patients with ALK translocations or EGFR mutations (Workman, 2020). However, in a phase 3 clinical trial, the combination of ganetespib plus the cytotoxic agent docetaxel showed no advantage over docetaxel alone in NSCLC (NCT01798485) (Ramalingam et al., 2014). Nevertheless, the role of HSP90 in mediating drug resistance via cancer evolution is being increasingly characterized (Courtin et al., 2016; Whitesell et al., 2014; Workman et al., 2016). Accordingly, HSP90 inhibitors could still be therapeutically relevant for inclusion in drug combinations for the treatment of appropriately selected cancer patient subpopulations (Workman, 2020). Moreover, the potential of HSP90 inhibitors to treat other diseases remains to be comprehensively explored, and recent research suggests that there are exciting opportunities, such as in Alzheimer disease (Inda et al., 2020), where the PU-AD has recently entered phase 1 clinical trials (NCT03935568), or in coronavirus infections, where ganetespib (ADX-1612) has recently entered phase 1 evaluation (https://www.aldeyra.com/pipeline-disease-areas/).

Among HSP90 clients, kinases are the most abundant protein family (Taipale et al., 2012), many of which are themselves drug targets. Dual inhibition of HSP90 and kinases could therefore be a very attractive strategy for cancer and potentially other diseases, and drug combinations have been suggested (Butler et al., 2015; Courtin et al., 2016; Schwartz et al., 2015; Wang et al., 2016). An alternative tactic is to identify small molecules that would inhibit both HSP90 and target kinases. Of note, there is already some evidence of HSP90-kinase cross-pharmacology. The recognition of the unconventional Bergerat fold enabling ATP binding in the GHKL ATPase/kinase superfamily (Dutta and Inouye, 2000)—which includes both HSP90 and histidine kinases—prompted the discovery that known kinase inhibitors could inhibit DNA gyrase B (Chène, 2002) and also that the HSP90 inhibitor radicicol inhibited the human 3-methyl-2-oxobutanoate dehydrogenase kinase (BCKDHK, half-maximal inhibitory concentration [IC_50_] = 635 nM) (Besant et al., 2002). Crystallographic evidence later showed that radicicol binds in the ATP-binding pocket of several kinases with low affinity and that the resorcinol moiety interacts via hydrogen bonding with the kinase hinge region of the bacterial sensor kinase PhoQ (K_d_ = 715 μM) (Guarnieri et al., 2008) and human pyruvate dehydrogenase kinases (IC_50_ = 230–400 μM) (Kato et al., 2007). A similar hinge-binding capacity has been demonstrated for other phenolic kinase inhibitor scaffolds (Caldwell et al., 2011). HSP90 inhibitor drug discovery projects, such as the one leading to the discovery of luminespib, included kinases in their off-target selectivity panels (Brough et al., 2008; Eccles et al., 2008). However, to our knowledge, none of the clinical HSP90 inhibitors was reported to inhibit any of the kinases tested.

Several years after the publication of the initial preclinical data on luminespib, an unrelated study analyzing kinase selectivity released a large biochemical screening dataset that included the original high-throughput screening hit (CCT018159, Figure 1) and a lead compound (VER-49009, Figure 1), which preceded the HSP90 inhibitor luminespib (Metz et al., 2011). Analysis of this dataset revealed that the hit and the lead compounds from the luminespib drug discovery project (Brough et al., 2008) show micromolar off-target inhibition of several kinases (Table S1) (Metz et al., 2011). More recently, a computational analysis of pharmacological databases uncovered these and a few other dual inhibitors of HSP90 and kinases (Anighoro et al., 2015). In the last few years four studies have rationally designed dual inhibitors of HSP90 together with PDKs (Tso et al., 2014), BCR-ABL (Wu et al., 2015), or ALK (Geng et al., 2018), and also a triple inhibitor of HSP90, JAKs, and HDAC (Yao et al., 2018), although the last two of these (Geng et al., 2018; Yao et al., 2018) have used pharmacophore linking rather than merging. Despite the above evidence of cross-pharmacology between HSP90 and kinases, the kinase polypharmacology of HSP90 clinical candidates has not been systematically characterized and represents a very interesting area that we felt should be explored in greater depth and scale. Moreover, the identification of off-target inhibition of kinases by the hit and lead compounds in the luminespib project offers an unprecedented opportunity to study how kinase polypharmacology evolved across drug discovery in the absence of an explicit selection pressure.

Here, we use computational and experimental methods to systematically explore at scale the kinase polypharmacology of representative clinical HSP90 inhibitors. We uncover the unique kinase polypharmacology of ganetespib and luminespib, the former inhibiting several kinases with nanomolar potency. We also demonstrate that kinase polypharmacology evolved during the discovery of luminespib and we recommend early assessment of polypharmacology in drug discovery projects so as to be aware of its potential adverse impact and also to unlock new multi-target drug design opportunities.

## Results

### *In silico* target profiling predicts differential kinase polypharmacology between clinical HSP90 inhibitors

We used three *in silico* target profiling methods to predict computationally the protein kinase off-targets of representative non-natural product clinical HSP90 inhibitors. The three methods were: a consensus of six ligand-based chemoinformatic methods integrated in the Chemotargets CLARITY platform (Vidal et al., 2011); the multinomial Naive Bayesian multi-category scikit-learn method available in ChEMBL (Mendez et al., 2018); and the Similarity Ensemble Approach (SEA) (Lounkine et al., 2012). All three methods use the common principle that structurally similar molecules are likely to bind to similar targets, but each one uses different similarity calculations and statistics. We selected the following representative synthetic HSP90 inhibitors from each of the chemical classes for *in silico* kinome profiling (Figure 1): three resorcinol derivatives (luminespib, ganetespib, and onalespib), two purine analogs (BIIB021 and Debio-0932), and the benzamide derivative SNX-2112 (Table S2).

Collectively, the methods correctly predicted most of the known interactions between the six selected HSP90 inhibitors and members of the HSP90 family (Table S2). In addition, two of the methods predicted previously unreported protein kinases as potential off-targets of several HSP90 inhibitors (Table 1). The computational methods implemented in CLARITY predicted that ganetespib and luminespib could both inhibit kinases off-target to different extents (Tables 1 and S2). The predictions for ganetespib had particularly high confidence (Table S2) and, in total, CLARITY predicted that this HSP90 inhibitor could inhibit 56 human kinases while luminespib might inhibit only two human kinases. In contrast, onalespib, the third resorcinol derivative, was not predicted to inhibit any kinase (Table S2).

**Table 1:** Comparison between the number of off-target kinases predicted for HSP90 inhibitors using three *in silico* target profiling methods and the number experimentally identified by *in vitro* kinome profiling employing a radiometric catalytic inhibition assay and applying a cutoff of >85% inhibition at 10 μM

		Method			
		Computational			Experimental
Chemical family	HSP90 inhibitor	CLARITY (Vidal et al., 2011)	ChEMBL (Mendez et al., 2018)	SEA (Keiser et al., 2009)	*In vitro* binding (KinomeSCAN) (Davis et al., 2011)
Resorcinol derivatives	luminespib	2	0	3	2
	ganetespib	58	0	0	21
	onalespib	0	0	0	0
Purine derivatives	Debio-0932	1	0	0	0
	BIIB021	0	0	0	0
Benzamide derivatives	SNX-2112	0	0	0	0

Note that luminespib and ganetespib were tested in a large kinome panel while the other HSP90 inhibitors were tested in a 16-kinase panel (Tables S1, S2, S3, S4, S5, and S6).

**Figure 2:**
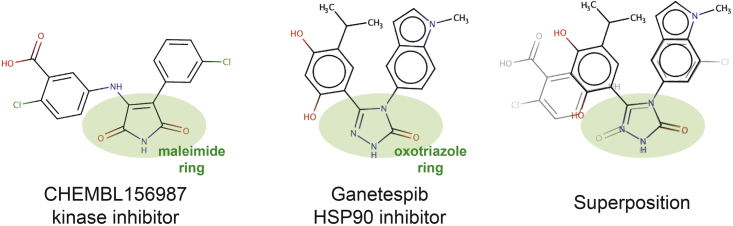
Chemical similarity between the kinase inhibitor CHEMBL156987 and ganetespib Their similar heterocycles are highlighted in green and superimposed on the right-hand side to highlight the complete overlap between the carbonyl and the nitrogen, which are likely to interact with the kinase hinge region in CHEMBL156987.

Inspection of the structure of CHEMBL156987 (Mendez et al., 2018), the compound with the highest chemical similarity to ganetespib that contributed to many of the kinase off-target predictions, showed a significant resemblance between the two compounds (Figure 2). As shown in Figure 2, the maleimide ring of CHEMBL15698, likely binding to the kinase hinge region (Witherington et al., 2003), is particularly similar to the triazolone ring of ganetespib. One carbonyl group and one nitrogen atom superimpose perfectly in both heterocycles (Figure 2), which could enable ganetespib to interact with kinases. In contrast, the core oxadiazole of luminespib lacks the N-H hydrogen bond donor common to the maleimide and triazolone rings. In addition, onalespib has no five-member heterocyclic ring and its tertiary amide linker confers a more extended conformation of the molecule compared with ganetesib and CHEMBL156987 (Figures 1 and 2). Therefore, it is reasonable to hypothesize that the capacity of their heterocycles to interact with the hinge region of kinases and the different conformations accessible to the various scaffolds could be driving the distinct predictions for the four resorcinol-derived HSP90 inhibitors analyzed. Interestingly, ChEMBL did not predict any kinase off-target for ganetespib, luminespib, or onalespib, but SEA predicted three kinases as potential off-targets of luminespib (Table S2). Overall, ganetespib and luminespib were most confidently predicted to interact with kinases off-target.

For the two purine analogs studied, CLARITY predicted only one kinase for Debio0932 (Tables 1 and S2), and neither ChEMBL nor SEA predicted any kinase off-targets (Table S2). Thus, the computational methods used do not suggest that the purine drug candidates are likely to inhibit kinases off-target.

None of the computational methods used predicted any kinase off-targets for the benzamide derivative SNX-2112 (Tables 1 and S2).

Overall, the computational methods we used predict that the capacity of HSP90 inhibitors to inhibit kinases off-target could vary greatly between the different chemical classes (Table 1), which could be associated particularly with the heterocyclic rings present in luminespib and ganetespib.

### *In vitro* kinome activity profiling uncovers differential polypharmacology between ganetespib and luminespib

To follow up the most promising computational predictions suggesting that luminespib and ganetespib could inhibit kinases off-target, we performed large-scale *in vitro* human protein kinome profiling of ganetespib and luminespib using Reaction Biology's HotSpot platform (Anastassiadis et al., 2011). The advantage of using this radiometric assay is that it measures inhibition of catalytic activity as opposed to the assays employed by other large-scale kinome profiling platforms that can report binding that may not translate into inhibition of catalytic activity (Ma et al., 2008). At the time of performing the experiments, the kinome panel used was the largest commercially available platform measuring catalytic activity and comprised 583 assays corresponding to 382 unique human kinases (74% of the human protein kinome) (Manning et al., 2002). In addition, 174 assays included mutated forms of kinases, 17 translocated products, and 10 other genomic aberrations (Table S3). The kinome profiling was performed at 10 μM concentration to identify both low- and high-potency kinase off-targets.

**Figure 3:**
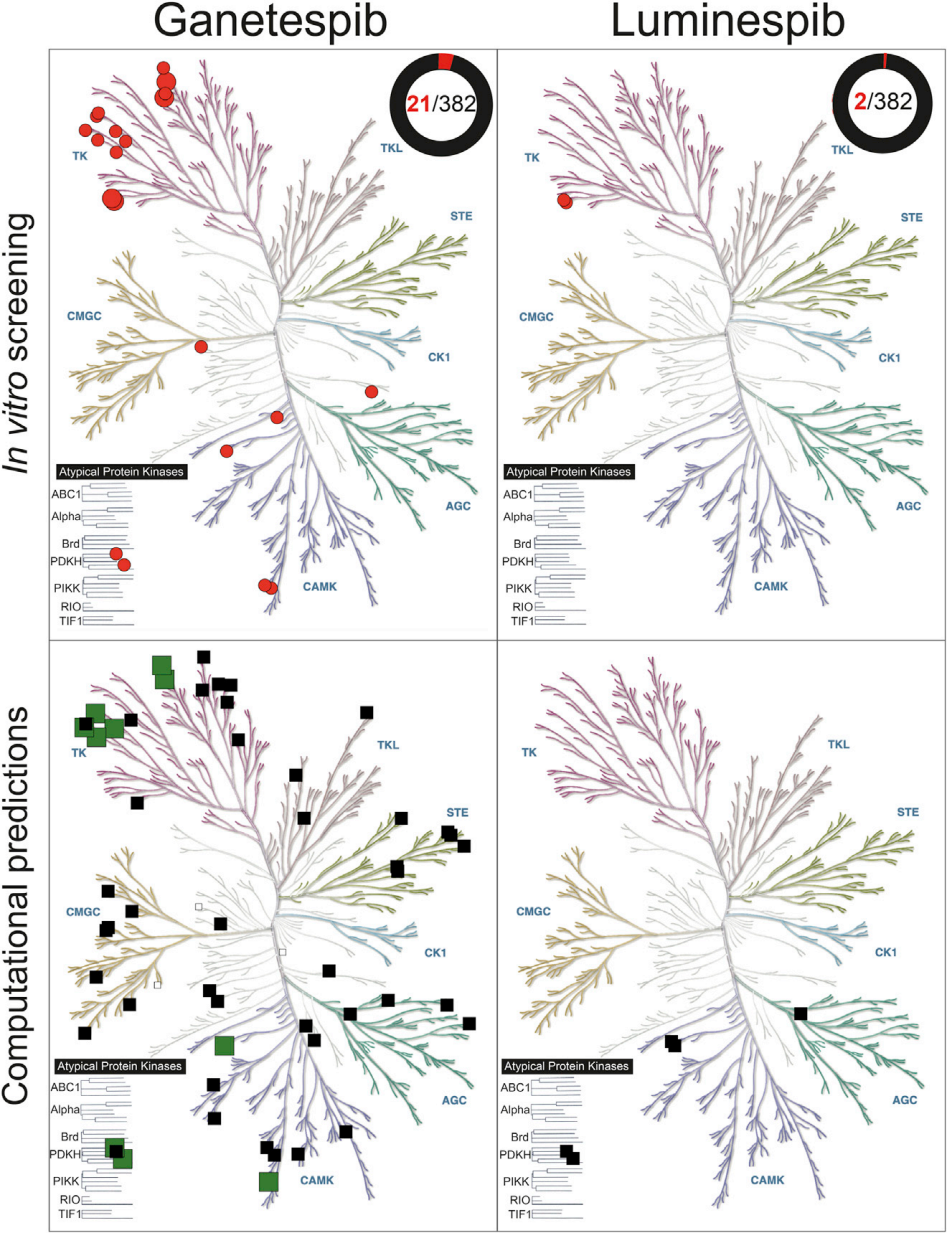
Experimental and computational results superimposed onto kinome trees for the HSP90 clinical inhibitors ganetespib (left) and luminespib (right) using KinMap The top panels display the experimental hits of the *in vitro* kinome screening using the catalytic inhibition assay, which are represented as red circles (see the STAR methods). Large red circles represent submicromolar interactions (IC_50_ < 1 μM) while medium red circles represent micromolar interactions either calculated from concentration-response experiments or expected based on the screening results (percent inhibition at 10 μM > 85% and percent inhibition at 1 μM < 85%). The circle in the top right corner of the two upper panels represents the portion of the screened kinome that was affected, emphasizing the high degree of kinome selectivity of both HSP90 inhibitors. The bottom panel merges the *in silico* predictions of the three computational methods used (see the Results and STAR methods) distinguishing between true positives (large green squares), false positives (medium black squares), and kinases, which could not be experimentally validated (small white squares).

The results of the *in vitro* kinome profiling validated our computational prediction that ganetespib and luminespib inhibit kinases off-target while uncovering significant differences between these two clinical HSP90 inhibitors. As illustrated in Figure 3 using Kin Maps (Eid et al., 2017), ganetespib inhibited 20 native kinases and also the fusion protein kinase TRKA-TFG (TRK-T3) by more than 85% at 10 μM, whereas luminespib inhibited only two native kinases (Table S3). Both drugs also inhibited several mutated forms of kinases. Given the variability associated with single-point high-concentration screens, we selected an 85% cutoff to increase the likelihood of the IC_50_ being lower than the tested concentration. Interestingly, the two kinases inhibited by luminespib, ABL1 and ABL2, were also inhibited by ganetespib with a higher percentage of inhibition at 10 μM. As illustrated in Figure 3, the kinase off-target activities of ganetespib are relatively widely distributed across the kinome tree, while the two kinases that luminespib was found to inhibit belong to the tyrosine kinase (TK) group.

Of the 58 human kinases that CLARITY predicted ganetespib or luminespib could inhibit, 10 were correctly predicted while 45 were false positives and 3 were not available in the selected kinase panel (Figure 3; Tables 1 and S2). Therefore, CLARITY had a sensitivity (recall) of 0.48 (10/21) when considering the kinase off-targets of ganetespib that showed >85% kinase inhibition *in vitro* at 10 μM while its precision was 0.17 (10/58). ChEMBL did not predict any kinase off-targets for luminespib or ganetespib and SEA predicted three kinases as potential off-targets of luminespib, none of which was validated *in vitro* if we apply the >85% cutoff. Therefore, the recall and precision were 0 for both methods (Table S2). Overall, the sensitivity and precision between these three computational methods was significantly different, despite their sharing of the same underlying principles. We have previously argued that the lack of data completeness, and the strong biases toward certain kinases, could contribute to explain the low precision of *in silico* prediction of polypharmacology (Antolin et al., 2020; Mestres et al., 2008; Workman et al., 2019). In this particular example, CLARITY's integration of several methods appears to be advantageous.

### Concentration-response determinations confirm ABL1, ABL2, DDR1, and TRKA-TFG as submicromolar off-targets of ganetespib

For further follow-up, we performed a secondary screening round at 1 μM using the same radiometric assay. We prioritized the 20 native kinases inhibited by more than 85% by ganetespib at 10 μM (Table S3) and also included the TRKA-TFG fusion. From these, only ABL2 and DDR1 were found to be inhibited by ganetespib by more than 85% at 1 μM (Figure 3; Table S3). Interestingly, both belong to the TK family (Figure 3).

**Figure 4:**
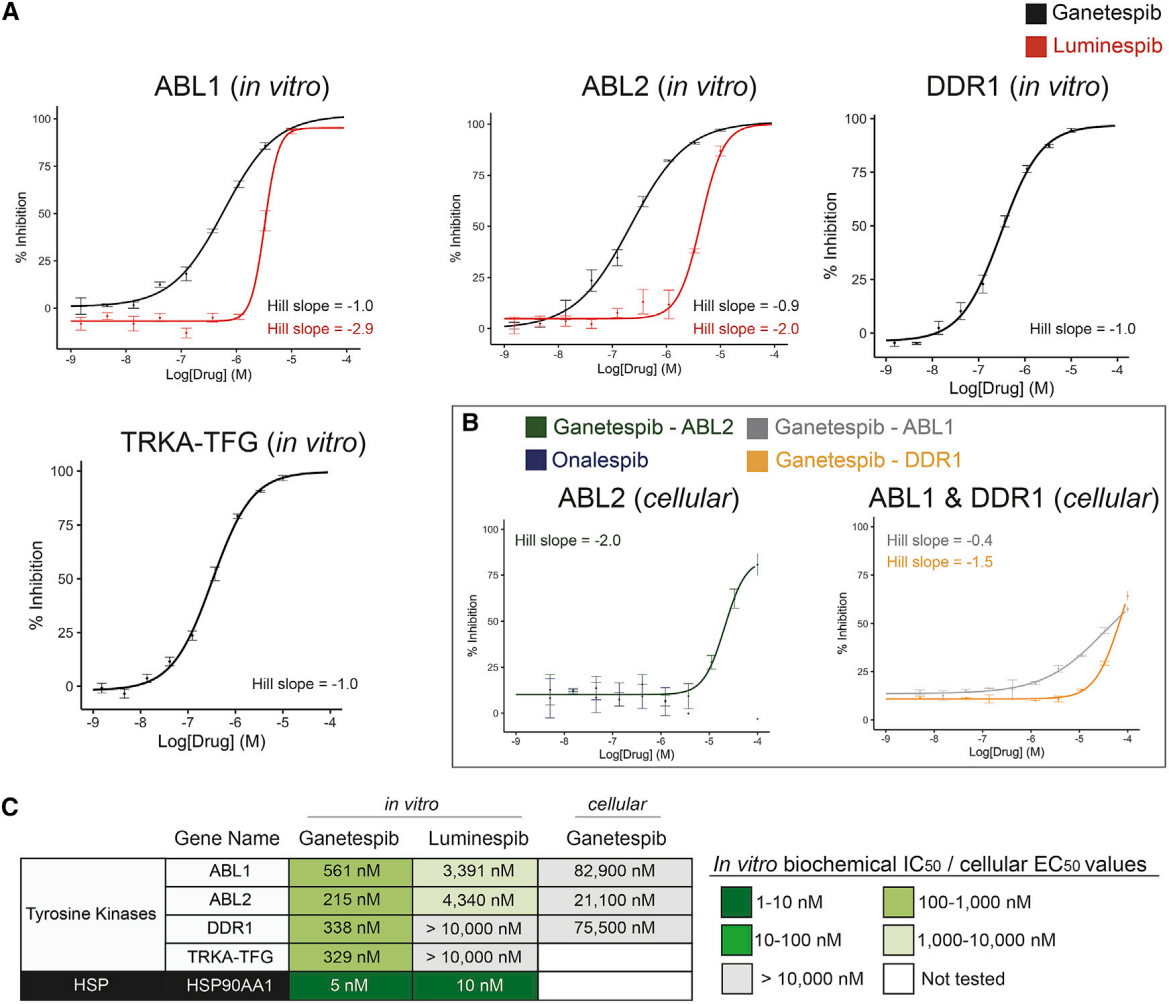
Concentration-response curves and IC_50_ values (n = 3) for the most potent kinase off-target interactions of luminespib and ganetespib (A) *In vitro* concentration-response curves for the interactions of ganetespib and luminespib with ABL1 and ABL2 and ganetespib with DDR1 and TRKA-TFG. (B) Cellular concentration-response curves of ganetespib and onalespib with ABL1, ABL2, and DDR1. (C) Table summarizing the calculated IC_50_ and EC_50_ values for the kinase off-targets of ganetespib and luminespib. HSP90AA1 IC_50_ average values obtained from ChEMBL are included for comparison (Table S1). Error bars in the concentration-response curves show the ranges observed on experimental repeats (Tables S4 and S6).

Next, we further explored the most potent interactions using the radiometric 10-point concentration-response inhibition assay in triplicate (Table S4). For luminespib, we selected the two native kinases whose activity was inhibited by more than 85% at 10 μM, namely, ABL1 and ABL2 (Table S3). For ganetespib, we selected ABL2 and DDR1, both inhibited by more than 85% at 1 μM. We also tested ganetespib against ABL1 and TRKA-TFG because of their potential therapeutic relevance, and since both of them were inhibited by more than 80% at 1 μM (Table S3). As can be observed in Figure 4, ganetespib exhibits submicromolar IC_50_ values for all of the four kinases tested, the most sensitive of which is ABL2 (IC_50_ = 215 nM). In contrast, luminespib exhibits low micromolar IC_50_ values for the two kinases tested, the most sensitive of which is ABL1 (IC_50_ = 3,391 nM).

Overall, the different kinase polypharmacology profiles of these two resorcinol HSP90 inhibitors has been experimentally confirmed, with ganetespib showing far greater kinase polypharmacology and more than 10-fold greater inhibitory potency against affected kinases than luminespib, which displays only very modest micromolar potency for only two kinases.

### Intracellular target engagement confirms binding of ganetespib to ABL1, ABL2, and DDR1 in transfected HEK293 cells

To demonstrate that the observed kinase polypharmacology occurred also in live cells, we used Reaction Biology's NanoBRET platform to determine intracellular target engagement for selected kinases. From the submicromolar off-targets identified in the biochemical radiometric catalytic assay (Figure 3), ABL1, ABL2, and DDR1 were available on the NanoBRET platform. We tested ganetespib against these three kinases. We also included onalespib as a negative control in the ABL2 assay. The results demonstrate concentration-responsive binding of ganetespib to the three kinases in this live HEK293 embryonic human kidney cell system (Table S6). The half-maximal effective concentration (EC_50_) values for the three interactions are in the 16–83 μM range (Figure 4). As expected, onalespib does not bind to ABL2 at the tested concentrations. Overall, these results further support the unique off-target inhibition of kinases by ganetespib and in particular reveal kinase engagement in live cells in the micromolar range.

### Kinase polypharmacology is not ubiquitous among HSP90 inhibitors

Having systematically characterized the kinase polypharmacology of ganetespib and luminespib, we wished to determine if this is a more general property of HSP90 inhibitors. Accordingly, we selected five additional representative HSP90 inhibitors, at least one from each chemical class, including both synthetic inhibitors and natural products. Thus, we chose the natural products geldanamycin and radicicol, the resorcinol derivative onalespib, the purine derivative Debio-0932, and the benzamide SNX-2112, the latter three being additional clinical candidates (Table S5). These five HSP90 inhibitors were screened at 10 μM concentration against the 15 kinases with >50% inhibition at 1 μM of ganetespib using the *in vitro* radiometric assay. The final 16-kinase panel used also included LYN B as the kinase with the greatest inhibition by luminespib, which is not inhibited by ganetespib. Interestingly, none of the additional HSP90 inhibitors tested displayed significant activity against any of the 16 kinases tested (Table S5). Thus, it appears that off-target kinase pharmacology seen with ganetespib and, to a lesser extent, luminespib, is not a general property of HSP90 inhibitors.

### Docking experiments to study HSP90-kinase cross-pharmacology at the atomic level

To study whether the unique capacity of HSP90 clinical inhibitors ganetespib and luminespib to inhibit kinases off-target was facilitated by their heterocyclic rings, we used molecular docking. We selected ABL1 as a representative kinase because it is the most sensitive off-target kinase for both inhibitors. We used the structure of ABL1 co-crystalized with tetrahydrostaurosporine, because tetrahydrostaurosporine is the inhibitor, co-crystalized with ABL1, that is most similar to ganetespib and its closest kinase inhibitor CHEMBL156987—which had enabled the off-target kinase prediction of ganetespib (Figure 2; STAR methods). We subsequently docked the five selected HSP90 inhibitors that were demonstrated not to inhibit ABL1 (Figure 1; Table S5) as well as ganetespib and luminespib. All dockings were performed using the Molecular Operating Environment (MOE) (see the STAR methods).

**Figure 5:**
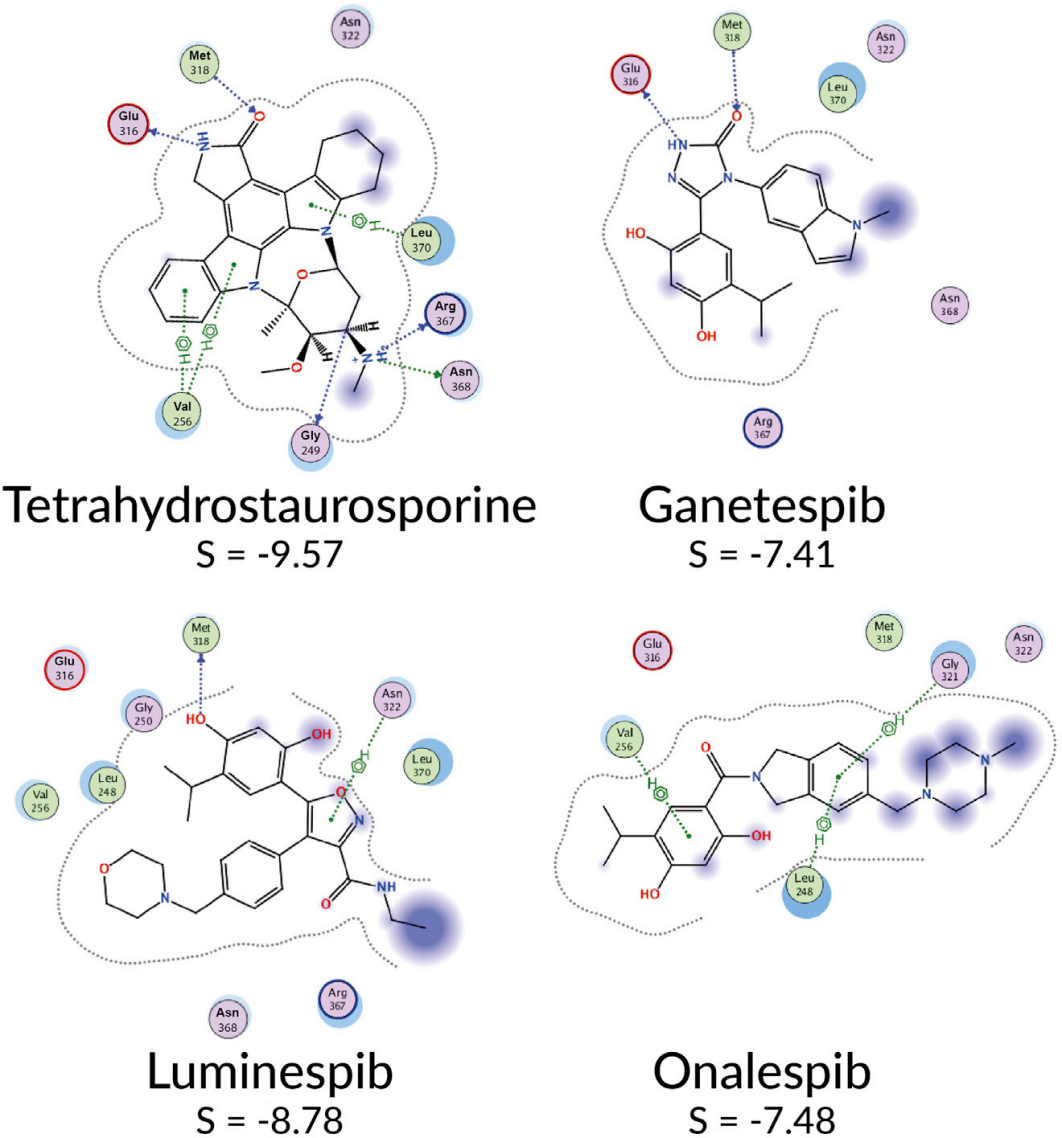
Analysis of ABL1-ligand interactions of different HSP90 inhibitors The co-crystalized ABL1 inhibitor tetrahydrostaurosporin and three clinical HSP90 inhibitors of the resorcinol family (ganetespib, luminespib, and onalespib) were docked using MOE and the best docking pose according to MOE score was analyzed using MOE's ligand interactions tool. The interaction diagrams for each of the four inhibitors are displayed highlighting only the protein residues interacting with at least one of the inhibitors. Ganetespib is the only HSP90 inhibitor predicted to mimic the double hydrogen bond that tetrahydrostaurosporin displays for interaction with the kinase hinge region.

The docking results were consistent with our hypothesis that the triazolone ring of ganetespib would be able to interact with the kinase hinge region, thus providing an explanation for the highest affinity of ganetespib for a larger number of kinases when compared with luminespib and the rest of HSP90 inhibitors studied. As illustrated in Figure 5, the interaction maps derived from the best docking poses for ganetespib, luminespib, and onalespib show how ganetespib is predicted to interact with the kinase hinge region, reproducing the double hydrogen bonding pattern of tetrahydrostaurosporine. Both tetrahydrostaurosporine and ganetespib are predicted to interact via hydrogen bonds with the ABL1 hinge region residues Met 318 and Glu 316. Luminespib's best binding pose predicted by docking places the isoxazole in a different area of the ABL1 ATP-binding site, although one of the hydroxyl groups of the resorcinol ring would still be able to form one hydrogen bond with the hinge residue Met 318. This same interaction of the resorcinol ring with Met 318 is maintained in radicicol's best pose (Figure S1) and is similar to the published crystal structures of radicicol with other kinases (Guarnieri et al., 2008; Kato et al., 2007). Onalespib, the third resorcinol derivative, is not predicted to make any hydrogen bond with the hinge region of ABL1. Thus, the resorcinol moiety appears not to be sufficient to achieve potent inhibition of the kinases tested, as illustrated by radicicol and onalespib—both inactive in the kinase panel tested. None of the rest of the HSP90 inhibitors from other chemical families that we found to be inactive against the 16-kinase panel (Table S5) were predicted to mimic the double hydrogen bond of tetrahydrostaurosporine with residues Met 318 and Glu 316 (Figure S1). However, the best docking poses predicted that some of the above inhibitors would be able to make hydrogen bonding interactions with hinge residues. The docking scores were poor predictors of ABL1 inhibition activity, in line with the ample evidence that docking performs better at predicting the binding pose of known inhibitors than at predicting binding affinity (Pantsar and Poso, 2018). Overall, our docking results support our hypothesis that the triazolone ring of ganetespib and its capacity to interact with the kinase hinge region could be responsible for driving its kinase polypharmacology.

### Evolution of kinase polypharmacology across luminespib drug discovery

Luminespib was discovered in a collaboration between our academic drug discovery team and Vernalis (Brough et al., 2008; Eccles et al., 2008). The chemical structures of the initial 3,4-diarylpyrazole resorcinol screening hit CCT018159 (Cheung et al., 2005; Sharp et al., 2007a), advanced lead compounds VER-49009 (Dymock et al., 2005) and VER-50589 (Sharp et al., 2007b), and the drug candidate itself (Figure 1), were disclosed and the compounds are commercially available. This offers an opportunity to study how kinase polypharmacology may have evolved across the drug discovery project in the absence of any explicit selection for kinase activity. Accordingly, we screened the hit compound and the two leads for kinase off-target inhibition using the 16-kinase panel and the same *in vitro* catalytic assay used previously. This panel includes 9 of the 14 kinase off-targets of luminespib inhibited by more than 50% at 10 μM and also the most sensitive off-targets of ganetespib that are not inhibited by luminespib (e.g., TRKA) to sample both off-targets that could be shared and those that could be different between luminespib and its precursor hit and lead compounds. Indeed, the screening did uncover off-targets that are in common and those that are distinct between the four compounds (Table S5).

**Figure 6:**
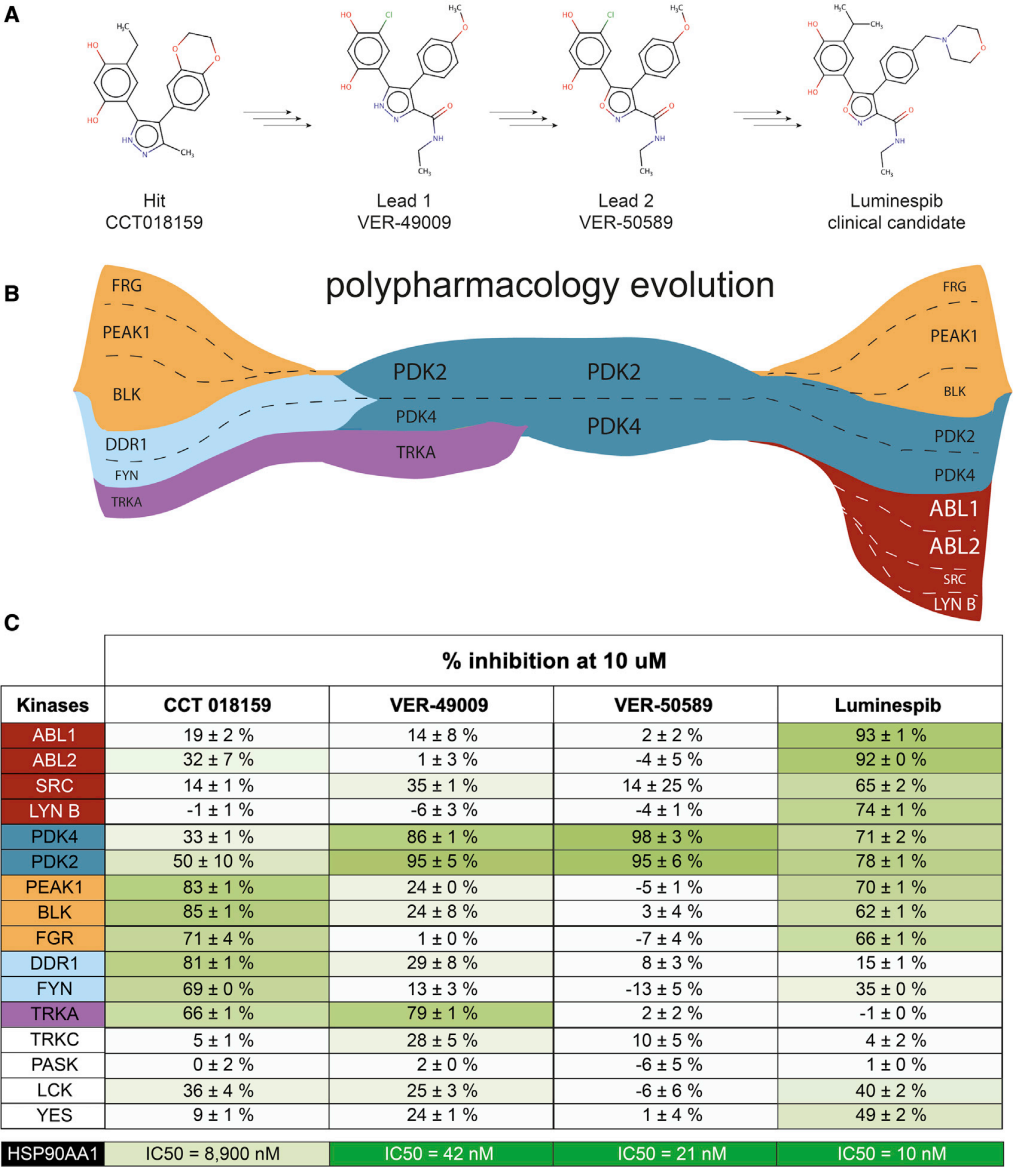
Evolution of kinase polypharmacology during the medicinal chemistry optimization of HSP90 inhibitors resulting in the discovery of luminespib (A) The chemical structures of the screening hit CCT018159, the two lead compounds VER-49009 and VER-50589, and the clinical candidate luminespib are displayed. (B) The evolutionary trajectories of the micromolar kinase off-targets of each of these compounds are displayed below the corresponding chemical structure (a >50% inhibition at 10 μM cutoff is used to determine that an off-target is inhibited; see panel C). The size of the kinase name is proportional to the extent of inhibition, larger size indicates greater inhibition (see panel C). Kinases are coloured to facilitate the identification of the ones shared by different compounds. (C) Table reporting median percent inhibition values for the four compounds against the 16-kinase panel at 10 μM ± standard deviation (n = 2) (green color intensity is proportional to percent inhibition, light green being less potent inhibition and darker green being highly potent inhibition) to further illustrate how kinase polypharmacology evolved across this particular drug discovery project. The IC_50_ against HSP90 for each compound is shown at the bottom for comparison.

As can be observed in Figure 6, polypharmacology evolved across luminespib's drug discovery history. Interestingly, the 4,5-diarylpyrazole screening hit CCT018159 and the final clinical candidate luminespib have more off-targets than the intermediate lead compounds VER-49009 and VER-50589 in the kinase panel used. Introduction of the 3-carboxamide substituent into the early lead 4,5-diaryl 3-carboxamido pyrazole VER-49009 significantly increased the off-target activity for PDK2/PDK4, which was maintained in subsequent HSP90 inhibitor optimization. Other kinase inhibitory activities of the hit were reduced by introduction of the 3-carboxamide substituent, itself an important contributor to affinity for HSP90. In addition, the change of five-membered heterocycle from the 4,5-diaryl 3-carboxamido pyrazole VER-49009 to the 4,5-diaryl 3-carboxamido isoxazoles VER-50589 and luminespib was associated with a large reduction of the TRKA off-target activity observed for the pyrazoles. The principal structural differences between the two 4,5-diaryl 3-carboxamido isoxazoles VER-50589 and luminespib are the introduction of a basic morpholino substituent and the change of the resorcinol chloro-substituent to the larger isopropyl group, while the core isoxazole amide scaffold is preserved. These changes in peripheral substitution are associated with an increase in the number of off-target kinases inhibited. The differential dependence of the off-target kinases on the presence of different functional groups could reflect the structure-activity relationships for each off-target kinase or may indicate that, in some cases, the scaffolds could adopt different binding poses between kinases, driven by different key interactions. All of the compounds have different polypharmacology profiles that evolved in non-obvious ways. For example, the hit CCT018159 and luminespib share >70% inhibition of PEAK1 that is not inhibited substantially by either of the intermediate lead compounds (<24% inhibition). The hit CCT018159 inhibits DDR1 (81% inhibition), a kinase that is inhibited by only 29% or less by the other compounds. Perhaps surprisingly, luminespib has a more similar off-target profile to the original screening hit CCT018159 than to the lead compounds VER-49009 and VER-50589 and inhibits four unique off-targets not shared with any of the hit and lead compounds. Overall, our results show that polypharmacology can significantly evolve during drug discovery and the off-target profile of the drug candidate can be different from that of the hit and lead compounds.

## Discussion

In this study, we have performed a comprehensive computational and experimental characterization of the protein kinase polypharmacology landscape of clinical HSP90 inhibitors. We have demonstrated that, in line with our computational predictions, off-target kinase pharmacology is a property of the clinical candidate ganetespib and, to a lesser extent, luminespib, another resorcinol clinical candidate (Figure 3). We show, however, that this off-target kinase profile is not an inherent property of all HSP90 inhibitors as the natural products geldanamycin and radicicol and the synthetic clinical candidates Debio-0932, onalespib and SNX-2112 were all inactive against the 16-kinase panel used to test them (Table S5). Ganetespib inhibits 21 native kinases *in vitro* with micromolar affinity, 4 of them with inhibition of catalytic activity below 1 μM. In contrast, luminespib inhibits only 2 kinases with micromolar affinity (Figures 3 and 4).

Of interest, from the 21 off-target kinases that were identified, 16 have been reported to be HSP90 client proteins, further emphasizing the potential relevance of their off-target inhibition (Table S3). It is worth noting that kinase inhibitors can lead to enhanced target degradation, and therefore reduced expression of the target kinase, by blocking recruitment of kinases to HSP90 by CDC37 (Polier et al., 2013). It is interesting to speculate that this effect could have indirectly favored the selection of dual HSP90-kinase inhibitors in cellular assays that measure client protein depletion and cell growth inhibition.

Importantly, our results reveal possible new opportunities for the rational design of dual HSP90-kinase inhibitors with potentially improved therapeutic properties and provide new lessons for better harnessing polypharmacology in drug discovery.

Of the three computational profiling methods used, two of these predicted kinases as off-targets of ganetespib and luminespib, despite there being significant differences in the sensitivity and precision of each method (Figure 3; Table S2). As we have recently shown in the case of PARP inhibitors (Antolin et al., 2020), these computational methods differ in specific results and may not predict the exact kinase off-targets, but collectively they correctly predict target classes and thus can usefully guide further experimentation. Despite the limitations, when used together, the methods were able to anticipate the kinase polypharmacology of ganetespib and luminespib as compared with other HSP90 inhibitors (Figure 3). Accordingly, we recommend the use of the different computational methods in conjunction to maximize recovery when predicting for potential off-target families. Although the methods successfully predicted the kinase class polypharmacology, they struggled to correctly predict the specific kinase off-targets. The main limitation is that all the methods use chemical similarity to compounds profiled in public medicinal chemistry databases, which are biased and only sparsely populated (Merget et al., 2017). Very few compounds are comprehensively profiled and most compound-target affinities have not been tested. As an illustration, we investigated the nearest neighbors for all the predictions reported by CLARITY (Table S2). Thirteen compounds dominated the predictions, being the nearest neighbors to the query compounds and being the best characterized among similar compounds. This makes the current prediction methods extremely vulnerable to data biases and data incompleteness. To alleviate this limitation, we urgently need a more unbiased dataset where representative scaffolds are comprehensively profiled for selectivity to reduce the sparsity of the data. As the matrix of chemotypes versus protein activities is increasingly populated, the methods will improve. It would be also important to thoroughly study the dependencies of the methods employed on the different chemical structure representation and statistical approaches used and also explore whether a better integration of methods or more advanced artificial intelligence approaches that do not rely on compound similarity could improve the sensitivity and precision of *in silico* polypharmacology prediction further.

Despite the clear usefulness of computational methods, our experimental kinome family-wide biochemical profiling using a radiometric catalytic assay identified a significant number of additional protein kinase off-targets *in vitro* that had not been predicted *in silico* (Figure 3). This indicates the importance of investigating experimental polypharmacology effects across a particular target family as a means to increase the number of identified off-targets. Note, however, that current kinome panels do not yet provide complete family coverage. It is therefore important that these panels continue to expand to facilitate the identification of a larger number of kinase off-targets in the future.

We have used the nanoBRET intracellular target engagement platform to demonstrate that ganetespib binds to ABL1, ABL2, and DDR1 in live cells in the 16–83 μM range (Figure 4). Differences between biochemical IC_50_ values and cellular EC_50_ values for kinase off-targets should be interpreted in light of different ATP-competition kinetics (Smyth and Collins, 2009). The >100-fold lower binding in cells as compared with the *in vitro* values that we observe (Figure 4) is consistent with the lower K_d,app_ (ATP-Mg) that has been reported for ABL1 (K_d_ = 37–99 μM) and ABL2 (K_d_ = 423 μM) using chemoproteomics (Becher et al., 2013). However, DDR1 (K_d_ > 1,000 μM) would be expected to exhibit much less of a drop in activity at typical intracellular concentrations of ATP (Smyth and Collins, 2009). These differences highlight the importance of measuring intracellular target engagement in cells as there are multiple factors (e.g., post-translational modifications) that are not well captured by *in vitro* assays and can affect compound activity in live cell systems. In any case, the modest EC_50_ values for kinase binding in cells caution against the potential clinical relevance of the kinase polypharmacology of ganetespib. To our knowledge, the unbound concentration of ganetespib in human plasma has not been reported, but the micromolar total plasma C_max_ values of ganetespib achieved in clinical studies (C_max,total_ = 6–12 μM) are already below the EC_50_ values that we obtained (Goyal et al., 2015). Note also, however, that ganetespib was found to inhibit several oncogenic fusion products of TRKA, that may be implicated in up to 1% of all solid tumors (Drilon et al., 2018), at nanomolar concentrations, but assays for these were not available in the NanoBRET target engagement platform. Therefore, it remains possible that off-target inhibition of TRKA-TFG by ganetespib could potentially be therapeutically relevant. We recommend that this should be investigated further in relevant cancer models to determine if ganetespib's kinase polypharmacology could be exploited therapeutically.

Although this study is focused on successfully determining the kinase inhibitory effects of HSP90 inhibitors, it is important to point out that the kinase inhibition seen is far weaker than the much more highly potent inhibition of HSP90—underlining the very high selectivity of all HSP90 inhibitors for the chaperone. For example, ganetespib is around 50-fold selective for HSP90 versus its most sensitive off-targets (Figure 4C; Tables S3, S4, and S5).

Understanding ganetespib's polypharmacology at the atomic level could be particularly important to facilitate the rational development of new HSP90-kinase multi-target inhibitors. Given the dual role of HSP90 in folding/stabilizing many kinases and in mediating drug resistance (Taipale et al., 2012; Wang et al., 2016; Workman et al., 2016), simultaneous inhibition of HSP90 and therapeutically important kinases could be an interesting strategy to prevent or delay the persistent problem of cancer drug resistance (Anighoro et al., 2014; Das et al., 2018; Schwartz et al., 2015; Workman et al., 2016). There is a high chemical similarity between ganetespib and its closest kinase inhibitor, CHEMBL156987. Both inhibitors have similar heterocyclic rings (Figure 2), which in the case of CHEMBL156987 was suspected by the researchers who discovered it to bind to the kinase hinge region of GSK3 (Witherington et al., 2003). Given the importance of the hinge region for kinase binding, we used docking to investigate whether the triazolone ring of ganetespib could be binding to the kinase hinge region. Indeed, docking results supported this hypothesis as the triazolone appears to be capable of reproducing the double hydrogen bond with two hinge residues observed in the cocrystal of tetrahydroxystaurosporin with ABL1 (Figure 5). Although the resorcinol moiety of radicicol has been shown in two crystal structures to interact with the kinase hinge region of PhoQ and pyruvate dehydrogenase kinases and confer modest binding (Guarnieri et al., 2008; Kato et al., 2007), we found that the resorcinol derivatives radicicol and onalespib were essentially inactive (<22% inhibition at 10 μM) in the kinase panel used, strengthening the hypothesis that the triazolone is the key moiety enabling the kinase polypharmacology of ganetespib. The best docking poses of the other HSP90 inhibitors studied here were not capable of reproducing this double hydrogen bond with ABL1 hinge region residues (Figures 5 and S1). Crystallographic determination of binding modes or more extensive structure-activity relationships for the off-target activities are required to confirm the docking predictions. Overall, the triazolone ring of ganetespib emerges as a potential privileged structure for simultaneous inhibition of HSP90 and kinases and a good starting point for future rational multi-target drug design endeavors.

Today, there is a growing interest in the rational design of multi-target drugs (Bolognesi, 2019; Hammam et al., 2017; Proschak et al., 2019). However, it is still very challenging to identify targets that can be simultaneously inhibited with a single compound and that are both therapeutically relevant for a specific disease (Proschak et al., 2019). Here, we have benefited from the availability through commercial vendors of the screening hit, advanced lead compounds, and clinical candidate to study how polypharmacology evolved during luminespib's drug discovery history. No explicit selection pressure for direct effects on kinases was applied as the kinase polypharmacology of these compounds was discovered *a posteriori*. To our surprise, both the clinical candidate luminespib and the screening hit CCT018159 display greater kinase polypharmacology than the intermediate lead compounds VER-49009 and VER-50589 (Figure 6; Table S5). This observation might be an important consideration when using lead compounds for proof-of-concept experiments. Although the 16-kinase panel employed is limited, we show how polypharmacology can evolve during a drug discovery project. All of the four compounds studied display a unique kinase polypharmacology profile and the kinase fingerprint of the drug candidate is not completely present in the screening hit or lead compounds (Figure 6). Albeit this is one single example, our findings suggest that many potentially interesting off-targets could be missed during drug discovery if only the clinical candidate is comprehensively profiled. Different kinase selectivity trajectories have also been observed during fragment growing of ATP-competitive kinase inhibitors, where minimal changes in the fragment can lead to new interactions with the target(s) that alter the kinase selectivity pattern of the fragment (Allen et al., 2014). We propose that a more systematic exploration of off-target pharmacology earlier in drug discovery campaigns could be helpful in the identification of multi-target drug design opportunities, or steering polypharmacology toward advantageous outcomes. Multi-target chemical series could potentially be developed in parallel with other chemical series aiming at single target inhibition and tested in follow-up phenotypic or efficacy experiments. For example, in the case of luminespib a dual HSP90-TRKA series inhibitor could potentially have been developed in parallel with a series aiming to design out any off-target kinase pharmacology. We also propose that computational prediction of polypharmacology, despite its limitations, might be very valuable at the earlier stages of drug discovery where comprehensive experimental profiling may not be cost-effective. Overall, the characterization of polypharmacology at earlier stages of drug discovery may unlock new multi-target drug design opportunities or avoid misleading findings in cellular assays.

In conclusion, characterization of polypharmacology is important because it provides the potential for dual- or multi-target inhibition by design, as well as helping to identify possible safety issues and unexplained cellular results in drug discovery. Our study demonstrates the unique kinase polypharmacology of the HSP90 inhibitors ganetespib and luminespib. In particular, despite its high selectivity for HSP90 versus off-target kinases, ganetespib does inhibit several human protein kinases with nanomolar affinity, a number of them being HSP90 clients. We have also demonstrated that ganetespib's polypharmacology translates into intracellular target engagement. Finally, our results demonstrate that polypharmacology can evolve unexpectedly during drug discovery. Therefore, we recommend the computational and experimental characterization of polypharmacology earlier in drug discovery projects, especially to harness untapped opportunities for multi-target drug design.

## Significance

**Polypharmacology affects the efficacy and safety of drugs. However, it is often explored only for the final drug candidate, limiting our capacity to harness polypharmacology in prospective multi-target drug design. Here, we use computational and experimental methods to systematically characterize the kinase polypharmacology landscape of the HSP90 inhibitors ganetespib and luminespib. We demonstrate that ganetespib and luminespib exhibit nanomolar inhibition of several kinases in biochemical assays and exhibit intracellular target engagement, while other HSP90 inhibitors do not show this polypharmacology. Moreover, we also demonstrate that polypharmacology can evolve during the drug discovery journey. Using the HSP90 inhibitor luminespib as an example, we illustrate how a screening hit and intermediate lead compounds can display different kinase polypharmacology compared with the clinical candidate. We recommend that polypharmacology should be explored earlier during drug discovery so as to be aware of its potential adverse impact and to unlock unanticipated multi-target drug design opportunities.**

## STAR★Methods

### Key resources table

**Table:** 

REAGENT OR RESOURCE	SOURCE	IDENTIFIER
**Chemicals, peptides, and recombinant proteins**		

Ganetespib (STA-9090)	Selleckchem	Catalog No. S1159
Luminespib (NVP-AUY922)	Selleckchem	Catalog No. S1069
Geldanamycin	Selleckchem	Catalog No. S2713
Radicicol	Tocris	Cat. No. 1589
SNX-2112 (PF-04928473)	Selleckchem	Catalog No. S2639
Onalespib (AT13387)	Selleckchem	Catalog No. S1163
Debio 0932	MedChem Express	Cat. No.: HY-13469
CCT 018159	Tocris	Cat. No. 2435
VER-49009	Selleckchem	Catalog No. S7458
VER-50589	Selleckchem	Catalog No. S7459

**Critical commercial assays**		

HotSpot kinase screening platform	Reaction Biology	HotSpot™
NanoBRET intracelular target engagement assays	Reaction Biology	NanoBRET

**Deposited data**		

ChEMBL27	Mendez et al., 2018	https://www.ebi.ac.uk/chembl/; RRID: SCR_014042
canSAR Black	Mitsopoulos et al., 2021	https://cansarblack.icr.ac.uk/; RRID: SCR_006794
PDB ID: 2HZ4	Cowan-Jacob et al., 2006	2HZ4; RRID: SCR_004312

**Experimental models: cell lines**		

HEK293 cells	ATCC	293 [HEK-293] (ATCC® CRL-1573™)

**Software and algorithms**		

CLARITY v3	Vidal et al., 2011	https://www.chemotargets.com
SEA	Keiser et al., 2009	https://sea.bkslab.org/
ChEMBL26 polypharmacology predictions	Mendez et al., 2018	https://www.ebi.ac.uk/chembl/
QuickPrep	MOE 2018.01	https://www.chemcomp.com/
Drc package (R)	Ritz et al., 2015	https://www.r-project.org/
Kin Map	Eid et al., 2017	http://www.kinhub.org/kinmap/

### Resource availability

#### Lead contact

Further information and requests for resources and reagents should be directed to and will be fulfilled by the lead contact, Albert A. Antolin (Albert.Antolin@icr.ac.uk).

#### Materials availability

This study did not generate new unique reagents.

#### Data and code availability

The published article includes all datasets generated during this study. Original data has been deposited in the canSAR Black knowledgebase and will be accessible to readers upon publication of this manuscript (Mitsopoulos et al., 2021). This article also analysed data from the publicly available resources ChEMBL (Mendez et al., 2018) (https://www.ebi.ac.uk/chembl/) and PDB (Velankar et al., 2018) (https://www.ebi.ac.uk/pdbe) using external software (see key resources table for details).

### Experimental model and subject details

#### Cell lines

HEK293 cells (female, human), which were established from primary embryonic human kidney, were obtained from ATCC. Cell lines were not authenticated in our hands, all experiments with cell lines were performed at the Contract Research Organization Reaction Biology. The cells are transfected using using EMEM + 10% FBS + 1% P/S medium. The assay itself is performed in Opti-MEM I reduced serum medium, without phenol red. EMEM is purchased from ATCC and Opti-MEM is purchased from ThermoFisher.

### Method details

#### *In silico* target profiling

The chemical structures of the HSP90 inhibitors were downloaded from ChEMBL (canonical SMILES) (Mendez et al., 2018). Three different *in silico* methods were used to predict the kinase off-targets of selected HSP90 clinical candidates, all exploiting the chemical similarity principle. The first method used was CLARITY (https://www.chemotargets.com), which computes a predefined consensus of six ligand-based chemoinformatic methods (Vidal et al., 2011). The second method employed was the Similarity Ensemble Approach (SEA) (http://sea.bkslab.org/) with default parameters (Lounkine et al., 2012). The third method selected was the similarity-based method available through the ChEMBL website (https://www.ebi.ac.uk/chembl/) (Mendez et al., 2018). Table S2 lists the predictions obtained from these three computational methods.

#### *In vitro* kinase radiometric assays

Reaction Biology’s HotSpot platform (http://www.reactionbiology.com) (Anastassiadis et al., 2011) was used at a compound concentration of 10 μM for kinome profiling and at 1 μM and/or 10-point concentration-response to validate the most potent hits. This radioisotope binding assay was designed to directly detect the true product without the use of modified substrates, coupling enzymes, or detection antibodies thus enabling to directly measure inhibition of catalytic activity. Test or control compounds were incubated with kinase, substrate, cofactors, and radioisotope-labeled ATP (32P-ɣ-ATP or 33P-ɣ-ATP). The reaction mixtures were then spotted onto filter papers, which bind the radioisotope-labeled catalytic product. Unreacted phosphate is removed via washing of the filter papers (Ma et al., 2008).

#### Intracellular target engagement kinase assays

We used Reaction Biology’s NanoBRET platform (http://www.reactionbiology.com) (Robers et al., 2015; Vasta et al., 2018) that employs a biophysical technique to quantitatively determine kinase inhibitor occupancy in live cells by a ligand in intact living cells using BRET with an optimized cell-permeable kinase tracer. The specificity of the BRET signal is dictated by the placement of NanoLuc on the chosen kinase and transfected into HEK293 cells, which were established from primary embryonic human kidney.

HEK293 cells were from ATCC. FuGENER HD Transfection Reagent, KinaseNanoLuc® fusion plasmids, Transfection Carrier DNA, NanoBRET™ Tracer and dilution buffer, NanoBRET™ Nano-Glo® Substrate, Extracellular NanoLuc® Inhibitor were from Promega. Onalespib was used as a negative control in the ABL2 assay and dasatinib was used as a positive control in all the assays.

HEK293 cells were transiently transfected with KinaseNanoLuc® Fusion Vector DNA by FuGENER HD Transfection Reagent. Test compounds were delivered into 384 well assay plate using an Echo 550 acoustic dispenser (Labcyte Inc, Sunnyvale, CA). Transfected cells were harvested and mixed with NanoBRET™ Tracer Reagent and dispensed into 384 well plates and incubated the plates at 37C in 5% CO_2_ cell culture incubator for 1 hour. The NanoBRET™ Nano-Glo® Substrate plus Extracellular NanoLuc® Inhibitor Solution were added into the wells of the assay plate and incubated for 2–3 minutes at room temperature. The donor emission wavelength (460 nm) and acceptor emission wavelength (600 nm) were measured in an EnVision plate reader. The BRET Ratio was calculated using the equation: BRET Ratio = [(Acceptor sample ÷ Donor sample) – (Acceptor no-tracer control ÷ Donor no-tracer control)].

#### Docking experiments

From the PDB, we selected the crystal structure corresponding to the wild type kinase domain ABL1 and co-crystalized with the most similar small molecule to ganetespib and its closest kinase inhibitor CHEMBL156987 that enabled the kinase off-target predictions (PDB: 2HZ4, Ligand: tetrahydrostaurosporine (4ST), (Cowan-Jacob et al., 2006)). The PDB file was prepared using the standard preparation method QuickPrep implemented in MOE 2018.01 (https://www.chemcomp.com/Products.htm). The mol files of the seven HSP90 inhibitors docked were downloaded from ChEMBL. The binding site was described using the cocrystalised ligand and docking was performed using standard variables for rigid docking. Best docking poses were analysed with MOE’s Ligand Interactions tool.

### Quantification and statistical analysis

#### IC_50_, EC_50_ and Hill slope determination

The IC_50_ and EC_50_ values (concentration causing a half-maximal inhibition of control specific activity) and Hill coefficients (nH) were determined by non-linear regression analysis of the inhibition curves generated with mean replicate values using Hill equation curve fitting (Y = D + [(A – D)/(1 + (C/C50)nH)], where Y = specific activity, D = minimum specific activity, A = maximum specific activity, C = compound concentration, C50 =IC50, and nH = slope factor). This analysis was performed using the R software and the package ‘drc’ (Ritz et al., 2015). In Figures 4 and 6, n represents the number of replicates and all the statistical details can be found in the Figure legends and the raw data in the Supplementary Tables.

## Supplemental information

Document S1. Figure S1

Table S1. Known target profile of selected HSP90 inhibitors, related to Figure 1

Table S2. Off-target predictions by the three computational methods used, related to Table 1 and Figure 2

Table S3. *In vitro* kinome profiling of ganetespib and luminespib, related to Figure 3

Table S4. Concentration-response curves of the most potent kinase off-targets of luminespib and ganetespib, related to Figure 4

Table S5. Kinase panel screening of representative HSP90 inhibitors, related to Figure 6

Table S6. Intracellular target engagement concentration-response curves, related to Figure 4

Document S2. Article plus supplemental information
