# Supplementary material for: Evolution of kinase polypharmacology across HSP90 drug discovery
Source: Cell Chem Biol. 2021 Oct 21;28(10):1433–1445.e3. doi: 10.1016/j.chembiol.2021.05.004 (PMC8550792; doi:10.1016/j.chembiol.2021.05.004)

Cell Chemical Biology, Volume *28*

Supplemental information

Evolution of kinase polypharmacology across HSP90 drug discovery

Albert A. Antolin, Paul A. Clarke, Ian Collins, Paul Workman, and Bissan Al-Lazikani

Supplemental Information

Figure S1. Protein-ligand interactions schemes for selected HSP90 inhibitors, related to Figure 5. **a** Docking pose with the top MOE score (S = -8.20) for SNX-2112 in ABL1 kinase. **b** Docking pose with the top MOE score (S = -8.02) for Debio-0932 in ABL1 kinase. **c** Docking pose with the top MOE score (S = -8.73) for geldanamycin in ABL1 kinase. **d** Docking pose with the top MOE score (S = -6.94) for radicicol in ABL1 kinase. The MOE ligand interaction tool was used to generate the schematic diagrams of protein-ligand interactions.

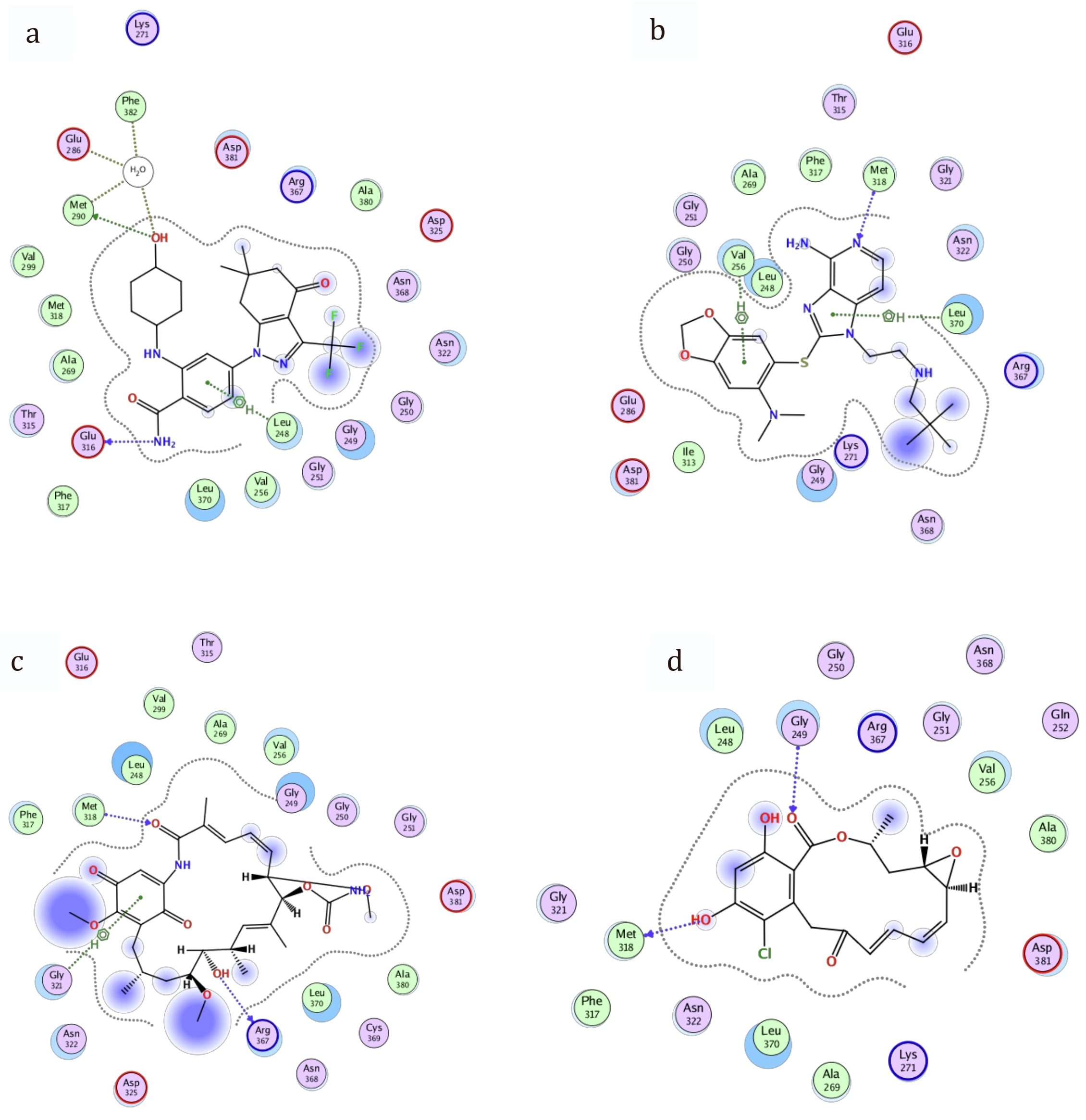

Supplement: Document S1. Figure S1 [file mmc1.pdf]
